# 4-Methyl-2-(2-nitro­benzene­sulfon­amido)­penta­noic acid

**DOI:** 10.1107/S1600536812033260

**Published:** 2012-07-28

**Authors:** Muhammad Nadeem Arshad, Muhammad Danish, Muhammad Nawaz Tahir, Savera Khalid, Abdullah M. Asiri

**Affiliations:** a The Center of Excellence for Advanced Materials Research (CEAMR), Faculty of Science, King Abdulaziz University, PO Box 80203, Jeddah 21589, Saudi Arabia; b Department of Chemistry, University of Gujrat, Gujrat 50700, Pakistan; c Department of Physics, University of Sargodha, Sargodha, Pakistan; d Department of Chemistry, Faculty of Science, King Abdulaziz University, PO Box 80203, Jeddah 21589, Saudi Arabia

## Abstract

In the title compound, C_12_H_16_N_2_O_6_S, the S atom adopts a distorted tetra­hedral geometry with an O—S—O angle of 119.76 (13)°. The nitro group is twisted by 35.34 (2)° with respect to the aromatic ring; it accepts an N—H⋯O hydrogen bond, resulting in a *S*(7) motif. In the crystal, N—H⋯O and O—H⋯O hydrogen bonds connect the mol­ecules into an infinite chain along the *a* axis. The methyl C atoms of the isopropyl group are disordered in a 1:1 ratio.

## Related literature

For a related structure, see: Arshad *et al.* (2010 ▶), For graph-set notation, see: Bernstein *et al.* (1995 ▶).
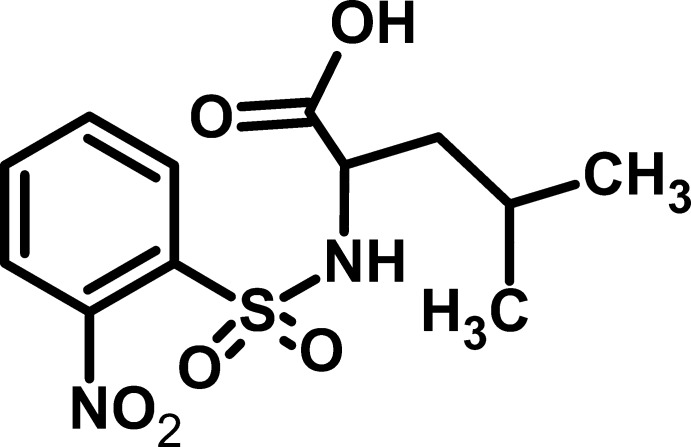


## Experimental

### 

#### Crystal data

C_12_H_16_N_2_O_6_S
*M* *_r_* = 316.33
Orthorhombic, 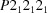

*a* = 6.9593 (5) Å
*b* = 10.7560 (8) Å
*c* = 20.8431 (14) Å
*V* = 1560.19 (19) Å^3^
*Z* = 4
Mo *K*α radiation
μ = 0.23 mm^−1^
*T* = 296 K
0.45 × 0.38 × 0.29 mm

#### Data collection

Bruker Kappa APEXII CCD diffractometer
Absorption correction: multi-scan (*SADABS*; Bruker, 2007 ▶) *T*_min_ = 0.902, *T*_max_ = 0.935
11203 measured reflections
2730 independent reflections
2029 reflections with *I* > 2σ(*I*)
*R*_int_ = 0.045

#### Refinement

*R*[*F* ^2^ > 2σ(*F* ^2^)] = 0.043
*wR*(*F* ^2^) = 0.090
*S* = 0.99
2730 reflections
212 parameters
10 restraints
H atoms treated by a mixture of independent and constrained refinement
Δρ_max_ = 0.15 e Å^−3^
Δρ_min_ = −0.20 e Å^−3^
Absolute structure: Flack (1983 ▶), 1117 Friedel pairs
Flack parameter: 0.07 (10)

### 

Data collection: *APEX2* (Bruker, 2007 ▶); cell refinement: *SAINT* (Bruker, 2007 ▶); data reduction: *SAINT*; program(s) used to solve structure: *SHELXS97* (Sheldrick, 2008 ▶); program(s) used to refine structure: *SHELXL97* (Sheldrick, 2008 ▶); molecular graphics: *PLATON* (Spek, 2009 ▶); software used to prepare material for publication: *WinGX* (Farrugia, 1999 ▶) and *PLATON*.

## Supplementary Material

Crystal structure: contains datablock(s) I, global. DOI: 10.1107/S1600536812033260/ng5283sup1.cif

Structure factors: contains datablock(s) I. DOI: 10.1107/S1600536812033260/ng5283Isup3.hkl

Supplementary material file. DOI: 10.1107/S1600536812033260/ng5283Isup3.cml

Additional supplementary materials:  crystallographic information; 3D view; checkCIF report

## Figures and Tables

**Table 1:** Hydrogen-bond geometry (Å, °)

*D*—H⋯*A*	*D*—H	H⋯*A*	*D*⋯*A*	*D*—H⋯*A*
N2—H2*N*⋯O2	0.85 (1)	2.34 (4)	2.937 (3)	128 (4)
O6—H6*O*⋯O5^i^	0.85 (1)	1.87 (2)	2.702 (3)	166 (5)
N2—H2*N*⋯O5^ii^	0.85 (1)	2.38 (2)	3.169 (3)	155 (4)

Symmetry codes: (i) 
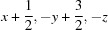
; (ii) 
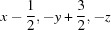
.

**Fig. 1.**
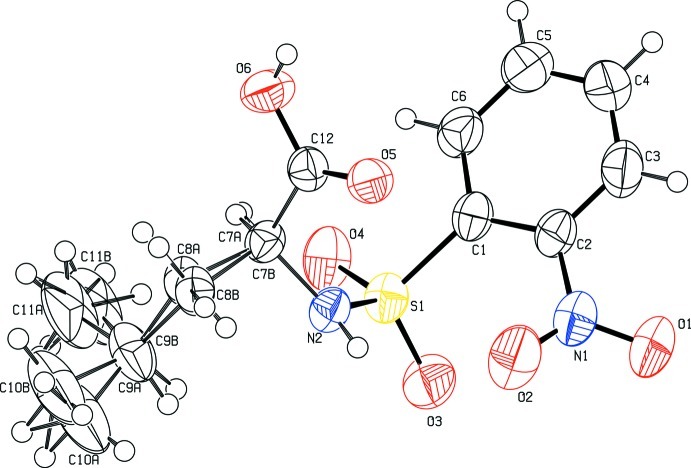
The labelled molecular structure of (I) with 50% displacement ellipsoids.

**Fig. 2.**
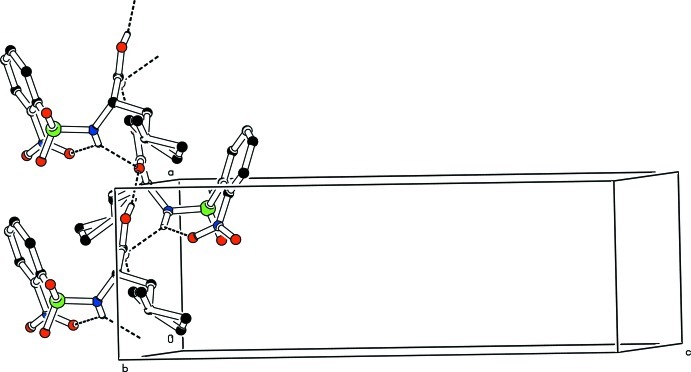
Unit cell packing showing hydrogen bonds, drawn using dashed lines. Hydrogen atoms not involved in hydrogen bonding have been omitted.

## supplementary crystallographic information

### Comment

In order to explore the structural behaviour of sulfonamide derived from amino acids (Arshad *et al.*, 2010), here we report the crystal structure of title compound.

The nitro group attached to aromatic ring is twisted at dihedral angle of 35.34 (2)°, with the maximum deviation from the two oxygen atoms being -0.532 (6) Å for O1 and 0.703 (5) Å for O2. An intramolecular N—H···O leads to the formation of a seven membered ring motif, *S*_1_^1^(7) (Bernstein *et al.*, 1995). The nitro group is oriented at an angle of 29.84 (6)° with respect to aromatic ring. Adjacent molecules are linked to form an infinite chain along *a* axis through O—H···O and N—H···O interactions (Table. 1, Fig. 2).

### Experimental

L-lucine (0.20 g, 0.089 mmole) dissolved in 5–10 mL distilled water was treated with sodium carbonate (1M) to a pH of 8–9. 2-Nitrobenzenesulphonyl chloride (0.117 g, 0.089 mmole) added within 3–5 min. The pH was adjusted by sodium carbonate (1M). Then, dilute HCl was added dropwise to result in a pH 2–3. The precipitate was filtered, washed with plenty of water and dried. Suitable crystals was obtained upon recrystalization in methanol.

### Refinement

All the C—H and H-atoms were positioned with idealized geometry with C—H = 0.93 Å for aromatic, C—H = 0.96 Å for methyl group and C—H = 0.97 Å for methylene, and were refined using a riding model with *U*_iso_(H) = 1.2 *U*_eq_(C) for aromatic and methylene and *U*_iso_(H) = 1.5 *U*_eq_(C) for methyl carbon atoms.

The N—H = 0.85 (1) and O—H = 0.85 (1) Å hydrogen atoms were located with difference map and were refined with *U*_iso_(H) = 1.2 *U*_eq_(N) and *U*_iso_(H) = 1.5 *U*_eq_(O).

Reaction does not affect the chirality of product, and the chirality is that of the reactant (L-Lucine).

The atoms C7—C11 were disordered over two positions with the occupancies of 0.50 for C7A—C11A and 0.50 for C7B—C11B. , The temperature factors of pairs of atoms were restrained to be identical. The C7a/C7b pair of atoms had the same site.

### Crystal data

**Table:**

C_12_H_16_N_2_O_6_S	*F*(000) = 664
*M**_r_* = 316.33	*D*_x_ = 1.347 Mg m^−^^3^
Orthorhombic, *P*2_1_2_1_2_1_	Mo *K*α radiation, λ = 0.71073 Å
Hall symbol: P 2ac 2ab	Cell parameters from 1789 reflections
*a* = 6.9593 (5) Å	θ = 2.7–19.5°
*b* = 10.7560 (8) Å	µ = 0.23 mm^−^^1^
*c* = 20.8431 (14) Å	*T* = 296 K
*V* = 1560.19 (19) Å^3^	Prismatic, colorless
*Z* = 4	0.45 × 0.38 × 0.29 mm

### Data collection

**Table:**

Bruker Kappa APEXII CCD diffractometer	2730 independent reflections
Radiation source: fine-focus sealed tube	2029 reflections with *I* > 2σ(*I*)
Graphite monochromator	*R*_int_ = 0.045
φ and ω scans	θ_max_ = 25.0°, θ_min_ = 2.0°
Absorption correction: multi-scan (*SADABS*; Bruker, 2007)	*h* = −8→8
*T*_min_ = 0.902, *T*_max_ = 0.935	*k* = −12→12
11203 measured reflections	*l* = −24→24

### Refinement

**Table:**

Refinement on *F*^2^	Secondary atom site location: difference Fourier map
Least-squares matrix: full	Hydrogen site location: inferred from neighbouring sites
*R*[*F*^2^ > 2σ(*F*^2^)] = 0.043	H atoms treated by a mixture of independent and constrained refinement
*wR*(*F*^2^) = 0.090	*w* = 1/[σ^2^(*F*_o_^2^) + (0.041*P*)^2^] where *P* = (*F*_o_^2^ + 2*F*_c_^2^)/3
*S* = 0.99	(Δ/σ)_max_ = 0.001
2730 reflections	Δρ_max_ = 0.15 e Å^−^^3^
212 parameters	Δρ_min_ = −0.20 e Å^−^^3^
10 restraints	Absolute structure: Flack (1983), 1117 Friedel pairs
Primary atom site location: structure-invariant direct methods	Flack parameter: 0.07 (10)

### Special details

**Table:**

Geometry. All esds (except the esd in the dihedral angle between two l.s. planes) are estimated using the full covariance matrix. The cell esds are taken into account individually in the estimation of esds in distances, angles and torsion angles; correlations between esds in cell parameters are only used when they are defined by crystal symmetry. An approximate (isotropic) treatment of cell esds is used for estimating esds involving l.s. planes.
Refinement. Refinement of F^2^ against ALL reflections. The weighted R-factor wR and goodness of fit S are based on F^2^, conventional R-factors R are based on F, with F set to zero for negative F^2^. The threshold expression of F^2^ > 2sigma(F^2^) is used only for calculating R-factors(gt) etc. and is not relevant to the choice of reflections for refinement. R-factors based on F^2^ are statistically about twice as large as those based on F, and R- factors based on ALL data will be even larger.

### Fractional atomic coordinates and isotropic or equivalent isotropic displacement parameters (Å^2^)

**Table:**

	*x*	*y*	*z*	*U*_iso_*/*U*_eq_	Occ. (<1)
S1	0.85210 (10)	0.49317 (7)	0.11950 (3)	0.0477 (2)	
O1	0.6834 (3)	0.8270 (3)	0.21732 (13)	0.0947 (9)	
N1	0.7618 (4)	0.7669 (3)	0.17531 (14)	0.0577 (7)	
C1	1.0039 (4)	0.5944 (3)	0.16432 (12)	0.0445 (7)	
O2	0.6943 (3)	0.7525 (2)	0.12226 (12)	0.0783 (7)	
N2	0.8391 (4)	0.5420 (2)	0.04664 (11)	0.0433 (6)	
C2	0.9481 (4)	0.7099 (3)	0.18997 (13)	0.0460 (8)	
O3	0.6652 (3)	0.5002 (2)	0.14685 (10)	0.0691 (6)	
C3	1.0679 (5)	0.7759 (3)	0.22962 (15)	0.0663 (10)	
H3	1.0269	0.8509	0.2471	0.080*	
O4	0.9507 (3)	0.37724 (17)	0.11794 (10)	0.0633 (6)	
C4	1.2476 (5)	0.7316 (4)	0.24342 (17)	0.0794 (12)	
H4	1.3294	0.7770	0.2698	0.095*	
O5	1.1024 (3)	0.73707 (19)	0.01558 (9)	0.0505 (6)	
C5	1.3060 (5)	0.6211 (4)	0.21847 (17)	0.0757 (11)	
H5	1.4283	0.5913	0.2278	0.091*	
O6	1.3184 (3)	0.5923 (2)	−0.00802 (12)	0.0610 (6)	
C6	1.1854 (5)	0.5525 (3)	0.17923 (14)	0.0569 (9)	
H6	1.2276	0.4769	0.1627	0.068*	
C12	1.1431 (4)	0.6294 (3)	0.00506 (12)	0.0429 (7)	
C7A	1.0001 (4)	0.5234 (2)	0.00245 (13)	0.0401 (7)	0.50
H7A	1.0676	0.4480	0.0161	0.048*	0.50
C8A	0.938 (3)	0.504 (2)	−0.0676 (7)	0.046 (3)	0.50
H81	0.8773	0.5793	−0.0830	0.056*	0.50
H82	1.0510	0.4890	−0.0936	0.056*	0.50
C9A	0.798 (5)	0.395 (3)	−0.0769 (14)	0.0651 (12)	0.50
H9A	0.7104	0.3945	−0.0400	0.078*	0.50
C10A	0.676 (4)	0.413 (4)	−0.1372 (12)	0.104 (7)	0.50
H10A	0.5886	0.4815	−0.1308	0.156*	0.50
H10B	0.6040	0.3390	−0.1457	0.156*	0.50
H10C	0.7580	0.4312	−0.1730	0.156*	0.50
C11A	0.903 (9)	0.272 (3)	−0.077 (3)	0.116 (7)	0.50
H11A	0.9924	0.2710	−0.1126	0.174*	0.50
H11B	0.8124	0.2058	−0.0824	0.174*	0.50
H11C	0.9716	0.2622	−0.0378	0.174*	0.50
C7B	1.0001 (4)	0.5234 (2)	0.00245 (13)	0.0401 (7)	0.50
H7B	1.0645	0.4444	0.0117	0.048*	0.50
C8B	0.910 (4)	0.520 (2)	−0.0647 (7)	0.046 (3)	0.50
H83	0.8243	0.5907	−0.0693	0.056*	0.50
H84	1.0109	0.5285	−0.0964	0.056*	0.50
C9B	0.797 (5)	0.401 (3)	−0.0783 (13)	0.0651 (12)	0.50
H9B	0.6792	0.4034	−0.0527	0.078*	0.50
C10B	0.739 (4)	0.398 (4)	−0.1495 (12)	0.104 (7)	0.50
H10D	0.6714	0.4726	−0.1601	0.156*	0.50
H10E	0.6568	0.3273	−0.1570	0.156*	0.50
H10F	0.8518	0.3907	−0.1756	0.156*	0.50
C11B	0.905 (9)	0.283 (3)	−0.061 (3)	0.116 (7)	0.50
H11D	1.0278	0.2832	−0.0812	0.174*	0.50
H11E	0.8321	0.2123	−0.0743	0.174*	0.50
H11F	0.9217	0.2803	−0.0149	0.174*	0.50
H2N	0.780 (5)	0.611 (2)	0.042 (2)	0.139*	
H6O	1.393 (6)	0.655 (3)	−0.013 (3)	0.174*	

### Atomic displacement parameters (Å^2^)

**Table:**

	*U*^11^	*U*^22^	*U*^33^	*U*^12^	*U*^13^	*U*^23^
S1	0.0575 (4)	0.0422 (4)	0.0435 (4)	−0.0031 (4)	0.0088 (4)	0.0032 (4)
O1	0.0808 (18)	0.107 (2)	0.096 (2)	0.0244 (16)	0.0092 (15)	−0.0496 (17)
N1	0.0688 (18)	0.0496 (18)	0.0548 (19)	0.0067 (14)	0.0099 (16)	−0.0070 (15)
C1	0.0542 (19)	0.0466 (19)	0.0328 (16)	0.0021 (16)	0.0081 (14)	0.0022 (14)
O2	0.0921 (16)	0.0889 (18)	0.0541 (14)	0.0387 (14)	−0.0074 (14)	0.0009 (14)
N2	0.0490 (14)	0.0458 (15)	0.0351 (13)	0.0012 (11)	0.0038 (12)	−0.0004 (11)
C2	0.0512 (17)	0.052 (2)	0.0349 (16)	0.0026 (16)	0.0075 (14)	0.0006 (15)
O3	0.0682 (14)	0.0802 (16)	0.0589 (13)	−0.0165 (15)	0.0231 (11)	−0.0023 (12)
C3	0.075 (2)	0.068 (2)	0.055 (2)	−0.004 (2)	0.0129 (18)	−0.019 (2)
O4	0.0946 (16)	0.0380 (13)	0.0574 (13)	0.0074 (10)	0.0061 (13)	0.0052 (11)
C4	0.062 (2)	0.106 (3)	0.070 (3)	−0.012 (2)	0.005 (2)	−0.034 (2)
O5	0.0464 (11)	0.0390 (13)	0.0661 (15)	−0.0011 (9)	0.0019 (10)	−0.0059 (11)
C5	0.058 (2)	0.108 (3)	0.061 (2)	0.012 (2)	−0.0014 (18)	−0.014 (2)
O6	0.0413 (13)	0.0466 (13)	0.0952 (17)	0.0033 (10)	0.0060 (12)	−0.0068 (13)
C6	0.058 (2)	0.071 (2)	0.0417 (18)	0.0103 (17)	0.0038 (16)	−0.0035 (16)
C12	0.0466 (16)	0.0431 (19)	0.0391 (17)	0.0025 (14)	−0.0021 (15)	−0.0025 (14)
C7A	0.0409 (15)	0.0398 (17)	0.0396 (15)	−0.0010 (13)	0.0078 (12)	−0.0005 (14)
C8A	0.053 (5)	0.042 (5)	0.044 (2)	−0.005 (4)	0.008 (3)	−0.009 (2)
C9A	0.085 (2)	0.058 (3)	0.052 (2)	−0.029 (2)	−0.0011 (19)	−0.007 (2)
C10A	0.143 (16)	0.115 (10)	0.055 (9)	−0.066 (13)	−0.013 (10)	−0.011 (6)
C11A	0.146 (4)	0.049 (5)	0.15 (2)	−0.017 (5)	−0.031 (13)	−0.046 (7)
C7B	0.0409 (15)	0.0398 (17)	0.0396 (15)	−0.0010 (13)	0.0078 (12)	−0.0005 (14)
C8B	0.053 (5)	0.042 (5)	0.044 (2)	−0.005 (4)	0.008 (3)	−0.009 (2)
C9B	0.085 (2)	0.058 (3)	0.052 (2)	−0.029 (2)	−0.0011 (19)	−0.007 (2)
C10B	0.143 (16)	0.115 (10)	0.055 (9)	−0.066 (13)	−0.013 (10)	−0.011 (6)
C11B	0.146 (4)	0.049 (5)	0.15 (2)	−0.017 (5)	−0.031 (13)	−0.046 (7)

### Geometric parameters (Å, º)

**Table:**

S1—O3	1.422 (2)	C8A—C9A	1.532 (12)
S1—O4	1.424 (2)	C8A—H81	0.9700
S1—N2	1.609 (2)	C8A—H82	0.9700
S1—C1	1.781 (3)	C9A—C11A	1.511 (13)
O1—N1	1.217 (3)	C9A—C10A	1.531 (13)
N1—O2	1.211 (3)	C9A—H9A	0.9800
N1—C2	1.466 (4)	C10A—H10A	0.9600
C1—C6	1.376 (4)	C10A—H10B	0.9600
C1—C2	1.408 (4)	C10A—H10C	0.9600
N2—C7A	1.464 (3)	C11A—H11A	0.9600
N2—H2N	0.850 (10)	C11A—H11B	0.9600
C2—C3	1.372 (4)	C11A—H11C	0.9600
C3—C4	1.369 (5)	C8B—C9B	1.530 (11)
C3—H3	0.9300	C8B—H83	0.9700
C4—C5	1.360 (5)	C8B—H84	0.9700
C4—H4	0.9300	C9B—C11B	1.513 (12)
O5—C12	1.212 (3)	C9B—C10B	1.538 (14)
C5—C6	1.385 (4)	C9B—H9B	0.9800
C5—H5	0.9300	C10B—H10D	0.9600
O6—C12	1.312 (3)	C10B—H10E	0.9600
O6—H6O	0.852 (10)	C10B—H10F	0.9600
C6—H6	0.9300	C11B—H11D	0.9600
C12—C7A	1.514 (4)	C11B—H11E	0.9600
C7A—C8A	1.538 (11)	C11B—H11F	0.9600
C7A—H7A	0.9800		
			
O3—S1—O4	119.76 (13)	N2—C7A—H7A	107.3
O3—S1—N2	108.02 (13)	C12—C7A—H7A	107.3
O4—S1—N2	106.94 (13)	C8A—C7A—H7A	107.3
O3—S1—C1	107.46 (13)	C9A—C8A—C7A	113.8 (14)
O4—S1—C1	105.15 (14)	C9A—C8A—H81	108.8
N2—S1—C1	109.20 (13)	C7A—C8A—H81	108.8
O2—N1—O1	123.4 (3)	C9A—C8A—H82	108.8
O2—N1—C2	118.7 (3)	C7A—C8A—H82	108.8
O1—N1—C2	118.0 (3)	H81—C8A—H82	107.7
C6—C1—C2	117.2 (3)	C11A—C9A—C10A	112.0 (15)
C6—C1—S1	117.6 (2)	C11A—C9A—C8A	111.0 (17)
C2—C1—S1	125.1 (2)	C10A—C9A—C8A	111.0 (15)
C7A—N2—S1	120.39 (19)	C11A—C9A—H9A	107.5
C7A—N2—H2N	115 (3)	C10A—C9A—H9A	107.5
S1—N2—H2N	114 (3)	C8A—C9A—H9A	107.5
C3—C2—C1	121.2 (3)	C9B—C8B—H83	108.9
C3—C2—N1	116.5 (3)	C9B—C8B—H84	108.9
C1—C2—N1	122.3 (3)	H83—C8B—H84	107.7
C4—C3—C2	120.1 (3)	C11B—C9B—C8B	113.7 (15)
C4—C3—H3	120.0	C11B—C9B—C10B	110.3 (16)
C2—C3—H3	120.0	C8B—C9B—C10B	109.5 (16)
C5—C4—C3	119.8 (4)	C11B—C9B—H9B	107.7
C5—C4—H4	120.1	C8B—C9B—H9B	107.7
C3—C4—H4	120.1	C10B—C9B—H9B	107.7
C4—C5—C6	120.7 (3)	C9B—C10B—H10D	109.5
C4—C5—H5	119.7	C9B—C10B—H10E	109.5
C6—C5—H5	119.7	H10D—C10B—H10E	109.5
C12—O6—H6O	111 (4)	C9B—C10B—H10F	109.5
C1—C6—C5	121.0 (3)	H10D—C10B—H10F	109.5
C1—C6—H6	119.5	H10E—C10B—H10F	109.5
C5—C6—H6	119.5	C9B—C11B—H11D	109.5
O5—C12—O6	123.0 (3)	C9B—C11B—H11E	109.5
O5—C12—C7A	124.9 (3)	H11D—C11B—H11E	109.5
O6—C12—C7A	112.0 (2)	C9B—C11B—H11F	109.5
N2—C7A—C12	112.2 (2)	H11D—C11B—H11F	109.5
N2—C7A—C8A	113.6 (10)	H11E—C11B—H11F	109.5
C12—C7A—C8A	108.9 (9)		
			
O3—S1—C1—C6	137.9 (2)	C1—C2—C3—C4	1.9 (5)
O4—S1—C1—C6	9.3 (3)	N1—C2—C3—C4	−177.2 (3)
N2—S1—C1—C6	−105.2 (2)	C2—C3—C4—C5	−0.9 (5)
O3—S1—C1—C2	−36.9 (3)	C3—C4—C5—C6	−0.1 (5)
O4—S1—C1—C2	−165.5 (2)	C2—C1—C6—C5	0.7 (4)
N2—S1—C1—C2	80.0 (3)	S1—C1—C6—C5	−174.5 (2)
O3—S1—N2—C7A	−168.2 (2)	C4—C5—C6—C1	0.2 (5)
O4—S1—N2—C7A	−38.0 (2)	S1—N2—C7A—C12	−88.8 (2)
C1—S1—N2—C7A	75.3 (2)	S1—N2—C7A—C8A	147.2 (10)
C6—C1—C2—C3	−1.8 (4)	O5—C12—C7A—N2	−32.7 (4)
S1—C1—C2—C3	173.0 (2)	O6—C12—C7A—N2	149.8 (2)
C6—C1—C2—N1	177.3 (3)	O5—C12—C7A—C8A	93.8 (11)
S1—C1—C2—N1	−7.9 (4)	O6—C12—C7A—C8A	−83.6 (11)
O2—N1—C2—C3	144.3 (3)	N2—C7A—C8A—C9A	−56 (2)
O1—N1—C2—C3	−35.4 (4)	C12—C7A—C8A—C9A	177.8 (18)
O2—N1—C2—C1	−34.8 (4)	C7A—C8A—C9A—C11A	−80 (3)
O1—N1—C2—C1	145.5 (3)	C7A—C8A—C9A—C10A	155 (2)

### Hydrogen-bond geometry (Å, º)

**Table:**

*D*—H···*A*	*D*—H	H···*A*	*D*···*A*	*D*—H···*A*
N2—H2*N*···O2	0.85 (1)	2.34 (4)	2.937 (3)	128 (4)
O6—H6*O*···O5^i^	0.85 (1)	1.87 (2)	2.702 (3)	166 (5)
N2—H2*N*···O5^ii^	0.85 (1)	2.38 (2)	3.169 (3)	155 (4)

Symmetry codes: (i) *x*+1/2, −*y*+3/2, −*z*; (ii) *x*−1/2, −*y*+3/2, −*z*.
